# Calmodulin and Its Binding Proteins in Parkinson’s Disease

**DOI:** 10.3390/ijms22063016

**Published:** 2021-03-16

**Authors:** Anastasiia Bohush, Wiesława Leśniak, Serge Weis, Anna Filipek

**Affiliations:** 1 Nencki Institute of Experimental Biology, Polish Academy of Sciences, 3 Pasteur Street, 02-093 Warsaw, Poland; a.bohush@nencki.edu.pl (A.B.); w.lesniak@nencki.edu.pl (W.L.); 2 Division of Neuropathology, Department of Pathology and Molecular Pathology, Neuromed Campus, Kepler University Hospital, Johannes Kepler University, A-4020 Linz, Austria; serge.weis@kepleruniklinikum.at

**Keywords:** Ca^2+^- signaling, Ca^2+^ homeostasis, calmodulin, calmodulin binding proteins, calcineurin, calmodulin kinase II, Parkinson’s disease

## Abstract

Parkinson’s disease (PD) is a neurodegenerative disorder that manifests with rest tremor, muscle rigidity and movement disturbances. At the microscopic level it is characterized by formation of specific intraneuronal inclusions, called Lewy bodies (LBs), and by a progressive loss of dopaminergic neurons in the striatum and substantia nigra. All living cells, among them neurons, rely on Ca^2+^ as a universal carrier of extracellular and intracellular signals that can initiate and control various cellular processes. Disturbances in Ca^2+^ homeostasis and dysfunction of Ca^2+^ signaling pathways may have serious consequences on cells and even result in cell death. Dopaminergic neurons are particularly sensitive to any changes in intracellular Ca^2+^ level. The best known and studied Ca^2+^ sensor in eukaryotic cells is calmodulin. Calmodulin binds Ca^2+^ with high affinity and regulates the activity of a plethora of proteins. In the brain, calmodulin and its binding proteins play a crucial role in regulation of the activity of synaptic proteins and in the maintenance of neuronal plasticity. Thus, any changes in activity of these proteins might be linked to the development and progression of neurodegenerative disorders including PD. This review aims to summarize published results regarding the role of calmodulin and its binding proteins in pathology and pathogenesis of PD.

## 1. Introduction

Parkinson’s disease (PD) is an age-related neurodegenerative disorder second in prevalence to Alzheimer disease (AD). PD manifests with rest tremor, muscle rigidity sometimes coupled with intense pain and with movement disturbances such as postural disabilities and gait disturbance. Non-motor features of PD include pain and sensory phenomena, anxiety and depression, autonomic dysfunction, cognitive impairment and dementia. It is a progressive disease, with symptoms aggravating with time. A characteristic histopathological feature of the affected brain areas of patients with PD or other synucleinopathies is the presence of specific inclusions such as Lewy bodies (LBs) and Lewy neurites (LNs) which contain mainly an aggregated form of α-synuclein [1]. The major characteristic of α-synuclein is its remarkable conformational plasticity [2]. The ability of α-synuclein to adopt toxic conformations might be due to the deficiency in the protein folding machinery that includes chaperones/co-chaperones or inadequate degradation of misfolded protein by the ubiquitin-proteasome system or the autophagy-lysosomal pathway. Thus, proper folding and degradation of α-synuclein are crucial factors for preventing accumulation of toxic intracellular inclusions [3]. Interestingly, it was found that mutated α-synuclein, present in patients with an early familial form of PD, is more prone to aggregation [4].

Appearance of motor symptoms in PD is attributed to loss of dopaminergic neurons in the striatum and substantia nigra. Usually, these symptoms develop when 70–80% of neurons are destroyed [5]. In turn, non-motor symptoms are a gradual consequence of progressive neurodegenerative changes in the brain [6]. There are multiple risk factors that increase the chance for developing PD. To them belong ageing or presence of mutations in genes encoding PD-related proteins such as α-synuclein, parkin, PINK1, DJ-1 or LRRK2 [7,8,9,10,11].

**Figure 1:**
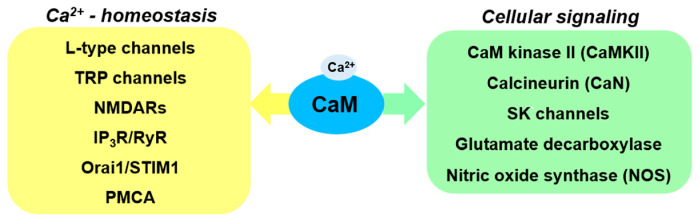
Selected calmodulin (CaM) targets involved in regulation of Ca^2+^-homeostasis and cellular signaling with potential role in Parkinson’s disease (PD).

Calmodulin (CaM), the best studied Ca^2+^-binding protein, is abundantly expressed in the brain. Particularly high levels of this protein are observed in postsynaptic membranes, postsynaptic densities and synaptic vesicles [12]. Of note, expression of CaM is up-regulated in response to the increase in intracellular Ca^2+^ concentration ([Ca^2+^]_i_). For instance, it was shown that CaM expression was higher in cells treated with rotenone, a known inhibitor of mitochondrial complex-I that increases [Ca^2+^]_i_ and triggers apoptosis [13]. CaM regulates the activity of a plethora of proteins, called calmodulin binding proteins (CaMBPs) [14]. In the brain, CaMBPs play a crucial role in the regulation of the activity of synaptic proteins and in the maintenance of neuronal plasticity. For that reason, CaMBPs have often been linked to the neurodegenerative disorders such as PD [15]. In this review, based on published data, we describe the potential involvement of CaM and selected CaMBPs (Figure 1) in pathology and pathogenesis of PD.

## 2. Ca^2+^ Signaling and CaM

All living cells rely on Ca^2+^ as a universal carrier of extracellular and intracellular signals that can ignite a plethora of cellular processes [16,17]. Ca^2+^ enters the cell along a steep concentration gradient, through various types of plasma membrane Ca^2+^ channels and can be removed from the cytoplasm against the gradient thanks to the action of the Na^+^/Ca^2+^ exchanger (NCX), plasma membrane (PMCA) and endoplasmic reticulum (SERCA) Ca^2+^-ATPases, and the mitochondrial Ca^2+^ uniporter (MCU). Ca^2+^ accumulated in the mitochondria and endoplasmic reticulum (ER) can be released into the cytoplasm via the mitochondrial Na^+^/Ca^2+^ exchanger (NCLX) and inositol-1,4,5-tris-phosphate receptor (IP_3_R) or ryanodine receptor (RyR) channels. Other types of channels and ATPases, for example in the Golgi or lysosomal membranes, are also part of the cellular Ca^2+^ transport system [16].

Dysfunction of Ca^2+^ signaling may have serious consequences and even result in cell death. Dopaminergic (DA) neurons are especially vulnerable to any disturbances in Ca^2+^ homeostasis because, due to their pacemaking activity mediated by the L-type Ca_v_1.3 channels, they experience regular Ca^2+^ fluxes but have poor Ca^2+^ buffering capacity [18]. Studies performed on animals indicate that neurons in the substantia nigra are much more vulnerable to neurodegeneration than, for example, striatal spiny neurons, in which the Ca_v_1.3 channels are only episodically activated [19]. Indeed, increased [Ca^2+^]_i_ in substantia nigra neurons was one of the earliest observations in PD brains [20]. Furthermore, it has now become obvious that many proteins implicated in PD are involved in controlling cellular Ca^2+^ fluxes and that their dysfunction caused by mutations results in serious disturbances in [Ca^2+^]_i_ [21]. For example, it was shown that mutations in α-synuclein enhanced formation of α-synuclein oligomers, which then integrated into the plasma membrane and facilitated Ca^2+^ entry into primary neurons and astrocytes [22]. Thus, disturbances in [Ca^2+^]_i_ observed in neurons of PD brain may then affect Ca^2+^ signaling pathways and cell functioning.

The major Ca^2+^ sensor in eukaryotic cells is CaM. It is a ubiquitous low molecular weight (16.7 kDa) protein, highly conserved within the plant and animal kingdom. In mammals, there are three genes encoding CaM, namely, *CALM1*, *CALM2* and *CALM3*, which differ only in the non-coding regions [23]. CaM belongs to the EF-hand protein superfamily [24]. It is composed of two, N- and C-terminal, globular domains or lobes, each containing two EF-hand motifs that bind Ca^2+^ with high affinity (10^−6^–10^−9^ M), linked by a highly flexible central linker domain. Upon Ca^2+^ binding, the helices in each EF-hand motif undergo a major rearrangement that substantially alters conformation of the two lobes and sets up the ground for a wide range of interactions. Moreover, due to high flexibility of the linker domain, the two lobes can interact with CaM-binding domains located within a variable distance, which contributes to the high number and versatility of CaMBPs [14]. Mechanistically, the interaction mediated by the two lobes of CaM can (1) link together two domains within a single protein molecule and change its conformation and activity, (2) bridge two protein molecules leading to dimerization or (3) bring together two different proteins building a protein scaffold [25]. Functionally, CaM binding may either positively or negatively modulate its target activity. Independently of the mode of interaction, CaM serves as a universal intermediary protein through which the Ca^2+^ signal is transmitted to numerous proteins and modulates a great number of cellular processes.

## 3. CaM-Regulated Ca^2+^ Homeostasis

CaM regulates several types of Ca^2+^ channels that provide Ca^2+^ influx into the cytoplasm. The list includes voltage-gated Ca^2+^ channels; *NMDA glutamate* receptors (NMDARs); Ca^2+^ permeable, non-selective transient receptor potential (TRP) channels; inositol 1,4,5-trisphosphate receptor (IP_3_R) channels that release Ca^2+^ from the ER and store operated Ca^2+^ (SOC) channels responsible for the refilling of the ER Ca^2+^ stores. The pore forming α1 subunit of the L-type voltage-gated Ca^2+^ channels has two CaM binding motifs. The one located in the C-terminal part is occupied by apo-CaM while the second, present in the N-terminal part of the molecule, interacts with the other lobe of CaM only in the presence of Ca^2+^. Upon Ca^2+^ entry through the channel, CaM brings together the N- and C-terminal regions of the α1 subunit and induces a conformational change that results in channel closure [25]. In the case of NMDAR, CaM binds in a Ca^2+^-dependent manner to the C-terminus of the NR1 subunit at two sites and induces inhibition of the Ca^2+^ flow. The mechanism may involve reversible dimerization of two NR1 subunits, whereby the two lobes of CaM would contact and bridge their C-termini [26]. Two CaM binding motifs were also identified in TRPV1 although the bridging mechanism has not been confirmed [27]. In the case of SOC channels, which are responsible for the major component of the Ca^2+^ influx in many excitable and non-excitable cells, CaM interacts with the C-terminal cytoplasmic domain of STIM1, a Ca^2+^ sensor protein located in the ER membrane, and disrupts its interaction with Orai1, the pore forming component of SOC in the plasma membrane [28]. Of note, an earlier work identified Orai1 as a CaM-binding protein [29], so the regulation might be, in fact, dual. Two CaM binding sites are also present in IP_3_R type 1. It is supposed that, in analogy to voltage-gated channels, CaM binds to one of these sites in an apo-form and, upon increase in [Ca^2+^]_i_, to the second one, bringing about a conformational change in the channel subunit and its deactivation [30]. As evidenced above, Ca^2+^-induced binding of CaM leads to inhibition of Ca^2+^ influx, a phenomenon known as Ca^2+^-dependent inactivation (CDI).

Regarding PD, many CaM-regulated Ca^2+^ channels seem to be implicated in this pathology. As mentioned above, in dopaminergic neurons of the substantia nigra, the L-type Ca^2+^ channels are responsible for the autonomous pacemaking Ca^2+^ influx. Of note, L-type Ca_v_1.3 channel expression was found to be higher in substantia nigra neurons of deceased PD patients [19]. Moreover, an increase in the level of Ca_v_1.2 and Ca_v_1.3 α1 subunits was also detected in the substantia nigra of MPTP-treated mice [31]. Isradipine, the L-type Ca^2+^ channel blocker, reduced motor impairment and prevented the loss of dopaminergic neurons in the striatum and substantia nigra of those animals [31]. Although the effect of isradipine was shown in animal models, recent data did not confirm the neuroprotective role of this drug in clinical studies. Such discrepancy might be due to different concentrations of isradipine used in these experiments [32].

Other L-type Ca^2+^ channels, Ca_v_1.2, were formerly considered to be functional only in excitable cells like dopaminergic neurons or muscle cells; however, recently, this type of channels has been found to function in microglial cells. Microglia in the brain play a major role in immune response and, thus, might be involved in neurodegeneration observed in PD. Activated microglia can exist in two stages, M1 and M2. The M1 stage, also called *neuroinflammatory*, when NOS (nitric oxide synthase) and NF-κB (nuclear factor kappa-light-chain-enhancer of activated B cells) pathways are activated*, *is responsible for** production of different pro-inflammatory factors such as *tumor necrosis factor α* (TNF-α), i*nterleukin 1*β (IL-1β), i*nterleukin* 6 (IL-6), reactive oxygen species (ROS) or nitric oxide (NO). In the M2 stage, called *neuroprotective, in which* production of i*nterleukin*-4 (IL-4)/i*nterleukin*-13 (IL-13) and i*nterleukin*-10 (IL-10)/*tumor growth factor* β (TGF-β) takes place, microglia facilitate phagocytosis of cell debris and misfolded proteins, promote tissue repair and support neuronal survival [33]. A recent study has demonstrated that Ca^2+^ antagonists enhanced the**neuroinflammatory** M1 stage and inhibited the *neuroprotective* M2 stage. Furthermore, these studies reported severe impairment of dopaminergic neurons accompanied by behavioral changes in microglia-specific Ca_v_1.2 knock-down mice treated with MPTP. These data prove detrimental effects of microglial Ca_v_1.2 blockade in PD [34].

Regarding the involvement of other CaM-regulated Ca^2+^ channels in PD, the data are still rather scarce. However, it was reported that dopamine depletion and L-DOPA treatment led to redistribution an altered ratio of NMDAR subunits in striatal synapses of both animal model and PD patient brains [35]. Moreover, in PD patients, a significant increase in NMDA-sensitive glutamate binding in the striatum was also observed [36]. Expression of another channel, TRPC1, which serves as a component of SOC channels, was reduced in the substantia nigra of mice that received intraperitoneal injections of MPPT and in rat pheochromocytoma PC12 cells incubated with MPP^+^ [37]. Overexpression of TRPC1 protected MPP^+^-treated PC12 cells against apoptosis and increased their survival while TRPC1 knock-down had a contrary effect, probably due to depletion of the ER Ca^2+^ store and induction of ER stress [38]. Other types of TRP channels, not involved in SOCE, also seem to be implicated in PD [39]. With regard to STIM1, the reports are so far contradictory. It was shown that STIM1 expression was unaltered in the substantia nigra of PD patients and that STIM1 silencing decreased the viability of human neuroblastoma SH-SY5Y cells [38]. However, STIM1 silencing in MPP^+^-treated PC12 cells resulted in increased cell viability and prevented mitochondrial dysfunction [40]. IP_3_Rs play an important role in maintaining Ca^2+^ homeostasis since in addition to Ca^2+^ release from ER, they also participate in Ca^2+^ transfer between ER and mitochondria [41]. The expression of IP_3_R type-1 in neurons of the cerebellum and motor cortex of rats with experimental hemiparkinsonism was found to be higher than in control rats [42]. However, it was shown that an inhibitor of RyR, 4-phenyl butyric acid (4-PBA), but not that of IP_3_R, was able to attenuate the increase in [Ca^2+^]_i_ observed in dopaminergic neurons treated with 6-hydroxydopamine (6-OHDA), an agent inducing parkinsonism [43]. The RyR inhibitor also protected cells from apoptosis.

Another CaM target that regulates Ca^2+^ homeostasis is the plasma membrane Ca^2+^-ATPase (PMCA). PMCA is an ATP-dependent ion pump that is responsible for Ca^2+^ efflux from the cytoplasm [44]. When [Ca^2+^]_i_ increases, PMCA is activated by CaM, acidic phospholipids and protein kinases and by other means, e.g., dimerization, and its affinity for Ca^2+^ increases (K_D_ ≈ 100–200 nM). CaM binds to the C-terminal autoinhibitory domain of PMCA and relieves the inhibition [45]. Regarding PD, it was demonstrated that in a cellular model of this disease, in which primary midbrain neurons were treated with MMP+, a significant downregulation of PMCA2 was observed [46]. In addition, downregulation of PMCA2 rendered the cells more vulnerable to MPP^+^-induced toxicity, whereas PMCA2 overexpression made these cells more resistant. However, to further elucidate the role of PMCA in PD additional studies, including animal models, are required.

As evidenced above, increased expression of Ca^2+^ channels and downregulation of PMCA are frequently reported in cellular and animal models of PD and both might contribute to the increased [Ca^2+^]_i_ observed in PD neurons. Thus, calmodulin, by providing a negative feedback regulation of Ca^2+^ channel activity and stimulating PMCA activity, appears as a crucial player in sustaining Ca^2+^ homeostasis that is so essential for neuronal survival.

## 4. CaM-Dependent Protein Kinase II and Its Substrates

**Figure 2:**
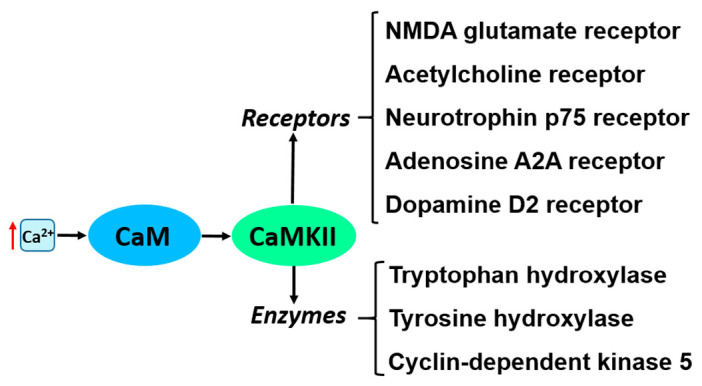
List of receptors and enzymes activated by Ca^2+^- CaM-CaMKII and potentially involved in PD. Red arrow indicates increase in intracellular Ca^2+^ concentration.

One of the important CaM binding proteins in the brain is CaM-dependent protein kinase II (CaMKII). At its basal state, CaMKII is locked in an inactive conformation through the binding of a regulatory segment to its substrate binding site. Upon the influx of Ca^2+^, CaM binds to CaMKII and induces its autophosphorylation/activation [47]. CaMKII is highly concentrated in the postsynaptic density (PSD), and its activation by CaM is a major event maintaining long-term potentiation (LTP), memory formation and neuronal excitability [48,49]. Molecular modeling studies revealed that formation of disulfide bridges in the CaMKII molecule, due to oxidative stress often prevailing in PD-damaged neurons, leads to the loss of CaM-CaMKII interaction and to defective Ca^2+_^ signaling in neurons [50]. CaMKII can also be inactivated following dephosphorylation by PP1 phosphatase [51]. Selected CaMKII substrates potentially involved in PD pathology are shown in Figure 2.

Experiments performed on mice treated with MPTP, a drug which selectively targets dopaminergic neurons in the substantia nigra, showed that reduction of CaMKII activity was associated with cognitive deficit and learning disability in these mice [52,53]. Interestingly, these findings positively correlated with the results showing a decrease in the level/activity of CaMKII substrate, tyrosine hydroxylase, a rate-limiting enzyme for dopamine synthesis [54]. Moreover, it was shown that CaMKII antagonist, KN-93, reduced autophosphorylation of the kinase and phosphorylation of tyrosine hydroxylase. Inhibition of these two proteins caused a decrease in L-DOPA-induced dyskinesia and extracellular dopamine efflux [55]. Notably, some other studies show that CaMKII activity is higher in a rat model of PD and that its inhibition reverses deficits in synaptic function and motor behavior [56]. CaMKII was also found to bind to dopamine D2 receptors in vitro and in rat striatal neurons in which these receptors are expressed at a high level. Interestingly, an increase in CaMKII-D2 receptor interaction in striatal neurons was observed in a rat model of PD after chronic administration of L-DOPA [57].

Multiple evidence points that there is a link between pathological changes of the cholinergic system (degeneration of cholinergic nuclei in the striatum) and motor symptoms in PD, such as L-DOPA-induced dyskinesia. CaM-CaMKII is involved in this pathway since it regulates, through phosphorylation, at least two components of this pathway, acetylcholine receptor and neurotrophin receptor p75 [58,59]. Interestingly, it was shown that inhibition of CaMKII resulted in loss of BDNF-induced inhibitory cholinergic transmission [59].

CaMKII phosphorylates and activates a key enzyme involved in serotonin synthesis, the tryptophan hydroxylase [60]. Interestingly, the activity of tryptophan hydroxylase was reduced in serotonergic neurons of PD patients [61], which then, most probably, resulted in non-motor psychiatric symptoms [62,63,64,65,66]. Based on these data, modulation of serotonin dependent neurotransmission by specific drugs might be considered in preclinical and clinical studies that aim at alleviating both motor and non-motor PD symptoms.

CaMKII also regulates the activity of adenosine A2A receptors (A2ARs) [67] that are involved in glutamate and dopamine release. A2ARs belong to the superfamily of G-protein-coupled receptors (GPCRs) which are abundantly expressed in the striatum, globus pallidus, and substantia nigra. Moreover, they interact and co-localize with dopamine receptors. Interestingly, it was shown previously that combination of simultaneous activation of dopaminergic receptors and inhibition of adenosine A2A receptors can improve mobility of PD patients. In agreement with this finding are data showing that a selective antagonist of adenosine A2AR, istradefylline (KW-6002), reversed the movement dysfunction and had a neuroprotective effect in animal models of PD [68]. Istradefylline improved mobility when used alone or when it was administered together with L-DOPA and dopamine receptor agonists. Long-term treatment with L-DOPA triggers side effects such as dyskinesia and abnormal involuntary movement in PD patients. Thus, to treat PD patients and reduce L-DOPA side effects adenosine A2AR antagonists in combination with L-DOPA can be applied. Quite recently, clinical trials on the potential use of istradefylline were completed and, what is important, it was found that such therapy can be applicable in both moderate and advanced stages of PD [69].

Another CaMKII substrate, deregulation of which might play an important role in PD, is cyclin-dependent kinase 5 (Cdk5). It was found that in a mouse model of PD Cdk5 activates inflammasomes, cytosolic multiprotein complexes responsible for the activation of pro-inflammatory responses [70]. Another study performed in a PD mouse model demonstrated that specific inhibition of Cdk5, by adeno-associated virus serotype-9 (AAV9) mediated Cdk5 inhibitory peptide (CIP), was protective against loss of dopaminergic neurons in the substantia nigra. Importantly, treatment with this inhibitor resulted in improved motor and anxiety-like symptoms in these mice [71]. Moreover, it was reported that inhibition of Cdk5 activity enhanced CaMKII autophosphorylation/activation in cultured neurons [72], which suggests that Cdk5 inhibitors can be considered as potential drugs in the treatment of PD symptoms. However, to justify a prospective therapeutic intervention aiming to normalize CaMKII activity and/or substrates in PD, further research is needed.

## 5. Involvement of Other CaM Binding Proteins in PD

Calcineurin (CaN) is a Ca^2+^ and CaM–regulated phosphatase. CaN is highly expressed in the mammalian brain, especially in neurons [73]. It is built of a catalytic subunit (CaNA) and a Ca^2+^-binding regulatory subunit (CaNB) [74]. CaNA contains an autoinhibitory domain and CaM-binding site. When [Ca^2+^]_i_ is low, the autoinhibitory domain masks the catalytic core and maintains CaN in an inactive state. Increase in [Ca^2+^]_i_ and binding of Ca^2+^ to CaM and to CaNB waives the inhibition and leads to rapid activation of the phosphatase. Neurodegeneration is characterized by activation of the CaN-NFAT signaling pathway and by pro-inflammatory gene expression [75,76]. Regarding PD, activation of CaN was found in brain at early stages of cognitive decline [77,78]. Similar changes in CaN activation were also observed in corresponding animal models of aging and neurodegeneration [79]. In turn, inhibition of CaN with the immunosuppressant drugs, tacrolimus and cyclosporine, protected brain cells from neurotoxicity in experimental models of these diseases [80,81], reduced neuroinflammation [82,83], improved the function of synapses [84], inhibited cognitive loss [85] and could even extend lifespan [86].

**Figure 3:**
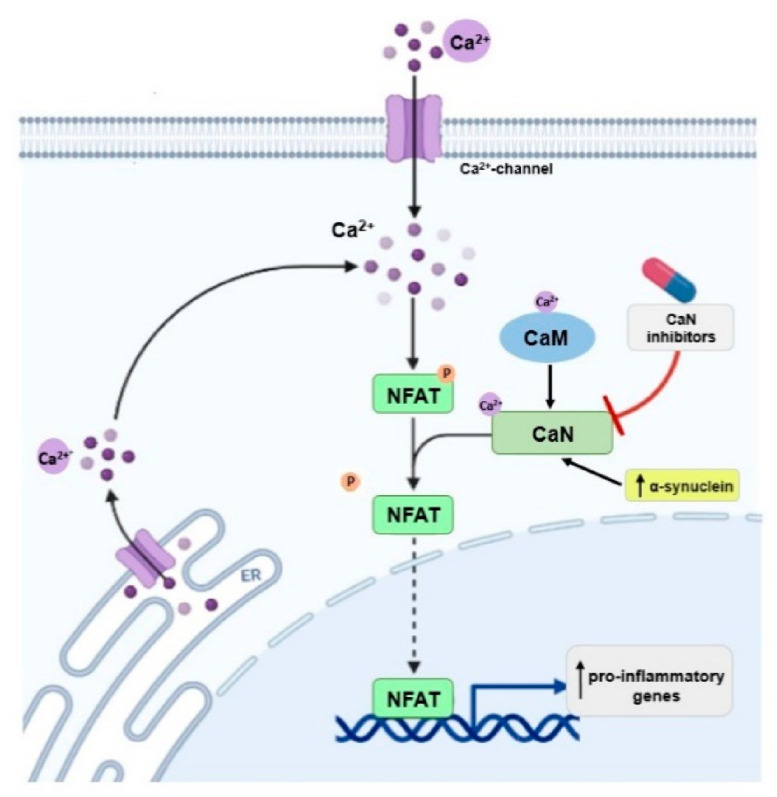
Possible involvement of α-synuclein in calcineurin (CaN) activation and enhanced expression of pro-inflammatory genes.

In vitro studies have shown that CaN interacts with α-synuclein, a main component of LBs and that this interaction is mediated by Ca^2+^-CaM [87]. Moreover, it was found that overexpression of α-synuclein activates the CaN-NFAT pathway in cell lines and dopaminergic neurons, whereas inhibition of this pathway protects dopaminergic neurons against α-synuclein-mediated toxicity [76]. This is in agreement with an in vivo study showing that overexpression of α-synuclein in mice significantly promoted CaN activity and subsequent nuclear translocation of NFAT transcription factor in dopaminergic neurons of midbrain [76]. However, another study indicated that inhibition of CaM and CaN, with genetic or pharmacological tools, shifts the α-synuclein-induced CaM-CaN cascade to a protective mode. This may have mechanistic implications for activity of CaN and may provide a therapeutic venue for the treatment of PD and other synucleinopathies [88] (Figure 3).

Small conductance Ca^2+^-activated K^+^ (SK) channels are another type of membrane proteins regulated by CaM. Their activity results in membrane hyperpolarization and reduced excitability and that is why they might serve as potential regulators of processes dependent on the membrane currents, including neurotransmitter release [89]. At low [Ca^2+^]_i_ apo-CaM binds to the C-terminal part of a channel subunit with one of its lobes while the other lobe binds to a site in another subunit upon increase in [Ca^2+^]_i_. Thus, CaM confers Ca^2+^ sensitivity to the SK channel gating mechanism and the resultant dimerization leads to channel activation [90]. Expression of SK channels, especially SK3 one, is high in dopaminergic neurons of the substantia nigra. This expression was found to be lower in rats infused with 6-OHDA into the striatum [91]. Blockade of SK channels showed many promising effects both in cellular and in animal models [89]. For example, in hemi-parkinsonian 6-OHDA lesion mouse model, SK channel inhibition by apamine protected nigral dopaminergic neurons and improved motor performance [91]. However, protective effects were also obtained with agonists of SK channels [89]. Adding to this discrepancy is the failure of a clinical study involving SK channel inhibition [92], which suggests that more information is needed before any therapeutical approaches can be considered.

An important enzyme activated by Ca^2+^-CaM is glutamate decarboxylase. It catalyzes α-decarboxylation of L-glutamic acid to γ-aminobutyric acid (GABA). Glutamate decarboxylase is widely distributed in eukaryotes as well as prokaryotes, where it plays different physiological functions. Regarding PD, a significant improvement in motor function was observed in PD patients after adeno-associated virus (AAV)-glutamic acid decarboxylase (GAD)-gene therapy. PET scans revealed a substantial reduction in thalamic metabolism that was restricted to the treated hemisphere, and a correlation between clinical motor scores and brain metabolism in the supplementary motor area. Moreover, AAV-GAD-gene therapy of the subthalamic nucleus of PD patients was safe and well tolerated [93].

One of the CaM binding proteins involved in a number of cellular processes is nitric oxide synthase (NOS). The neuronal isoform of NOS (nNOS) is expressed in both immature and mature neurons and requires Ca^2+^-CaM for its activity [94,95,96]. CaM functions as a molecular switch, allowing electron transport from the C-terminal reductase domain of NOS to its heme-containing N-terminal domain. Regarding PD, high levels of nNOS and inducible NOS (iNOS) expression were observed in the substantia nigra and striatum of PD patients and of experimental PD models [97]. Some other studies show that the nNOS knockout mice are more resistant to MPTP-induced neurotoxicity compared with wild-type animals [98]. This was confirmed by using the nNOS inhibitor, 7-nitroindazole. Applying this agent protected neuronal cells against MPTP-induced neurotoxicity in animal models [99,100]. Moreover, an overexpression of nNOS was reported in basal ganglia and in the respiratory burst of circulating neutrophils of PD patients; at the same time, a significant increase in NO production and protein tyrosine nitration were observed [101]. Based on these data it can be concluded that nNOS plays a key role in the pathogenesis of PD and that antioxidant and anti-inflammatory agents could be considered for treatment of this disease.

## 6. Conclusions

Parkinson’s disease (PD) is an age-related progressive neurodegenerative disorder with symptoms aggravating with time. Appearance of PD symptoms is attributed to the loss of dopaminergic neurons in the striatum and substantia nigra. Neuronal cell death may be ascribed to increased [Ca^2+^]_i_, which is commonly observed in cells in various areas of PD brain. Upon physiological increase in [Ca^2+^]_i_, a major Ca^2+^ sensor, CaM, interacts with many CaMBPs, which are involved in Ca^2+^-homeostasis, intracellular Ca^2+^-signaling pathways and other cellular processes. To CaMBPs belong different types of Ca^2+^channels, plasma membrane Ca^2+^-ATPase and many enzymes, of which a kinase, CaMKII, and a phosphatase, CaN, are of most importance. It might be assumed that under sustained Ca^2+^- signaling, the activity of CaM is markedly enhanced and may lead to excessive activation of CaMBPs with harmful consequences for different cell types, including neurons. Indeed, experimental evidence suggests that, in most cases, inhibition of CaMBPs, by various specific drugs brings about beneficial effects both in animal models of the disease and in PD patients.

As it was described in this review, published data show that inhibition of CaM-regulated proteins such as L-type Ca^2+^ channels, RyR receptors, SK channels or NOS may protect dopaminergic neurons from apoptosis and improve motor function in mouse model of PD. Moreover, it was shown that CaMKII antagonist caused a decrease in dyskinesia and extracellular dopamine efflux in an experimentally induced PD model. Moreover, drugs that inhibited CaMKII binding partners, to which belong tryptophan hydroxylase, adenosine A2A receptor or Cdk5 kinase had a positive effect on dopaminergic cell survival. Similarly, inhibition of CaM-activated phosphatase, CaN, protected brain cells from neurotoxicity, neuroinflammation and improved cognitive function in experimental models of PD and other neurodegenerative diseases. All CaM binding proteins described in this work and their function in norm and PD pathology are summarized in Table 1. Altogether, available results indicate that modulation of activity of various CaMBPs might be considered in designing new therapies to treat PD patients.

## Figures and Tables

**Table 1:** Calmodulin binding proteins (CaMBPs) and their function in norm and PD.

CaMBP	Norm	PD Pathology	Effect of Modification of Protein Level/Activity
**L-type channels**	Ca^2+^ influx	Increased expression in substantia nigra neurons of deceased PD patients [19].	Ca_V_1.2 channel blocker protects dopaminergic neurons exposed to rotenone or MPTP [19,31]. Ca_V_1.2 channel knock-down in microglia impairs dopaminergic neurons in MPTP-treated mice [34].
**TRP channels**	Ca^2+^ influx	Reduced expression in the substantia nigra neurons of PD model [37].	Overexpression of TRPC1 protects MPP^+^-treated PC12 cells against apoptosis and increases their survival [38].
**NMDARs**	L-glutamate receptors, mediate Ca^2+^ influx, role in learning and memory.	Increase in NMDA-sensitive glutamate binding in the striatum of PD patients [36]. Redistribution and altered ratio of NMDAR subunits in striatal synapses of both animal model and PD patient [35].	ND
**IP_3_R/RyR**	Ca^2+^ release from ER and Ca^2+^ transfer from ER to mitochondria.	Increased expression of IP_3_R in cerebellum and motor cortex of rat PD model [42].	RyR blockade attenuates Ca^2+^ overload, preserves excitability and protects dopaminergic neurons from apoptosis in animal and cellular models of PD [43].
**Orai1/STIM1**	*Replenishment* of ER Ca^2+^ stores.	STIM1 expression is unaltered in the substantia nigra of PD patients [38].	STIM1 silencing decreases viability of human neuroblastoma SH-SY5Y cells [38]. STIM1 silencing in MPP^+^-treated PC12 cells prevents mitochondrial dysfunction and improves cell viability [40].
**PMCA**	Ca^2+^ efflux	Decreased PMCA2 expression in a cellular PD model [46].	PMCA2 downregulation sensitizes cells to, and upregulation protects from, MPP+ toxicity [46].
**CaMKII**	Maintaining long-term potentiation (LTP), memory formation and neuronal excitability.	CaMKII activity is higher in a rat model of PD [56].	CaMKII inhibition reverses deficits in synaptic function and motor behavior in a rat model of PD [56]. Reduction of CaMKII activity is associated with cognitive deficit and learning disability in mouse model of PD [52,53].
	Involvement in dopamine synthesis [54].	Increased interaction of CaMKII-D2 receptor in striatal neurons of a rat model of PD after chronic administration of L-DOPA [57].	CaMKII inhibition reduces of tyrosine hydroxylase phosphorylation [55].
	CaMKII mediates cholinergic system by regulation of acetylcholine receptor and neurotrophin receptor p75 [58,59].	ND	Inhibition of CaMKII results in loss of BDNF-induced inhibitory cholinergic transmission [59].
	Activation of tryptophan hydroxylase, a key enzyme involved in serotonin synthesis [60].	Activity of tryptophan hydroxylase is reduced in serotonergic neurons of PD patients [61].	ND
	Regulation of A2AR activity [67].	ND	Inhibition of A2ARs reverses movement dysfunction and is neuroprotective in animal models of PD [68].
**CaN**	Maintaining neuronal plasticity, long-term potentiation (LTP), memory formation.	CaN is activated in brain at early stages of cognitive decline [77,78].	Inhibition of CaN protects brain cells from neurotoxicity [80,81], reduces neuroinflammation [82,83], improves the function of synapses [84], inhibits cognitive loss [85], and could extend lifespan [86].
		CaN interacts with α-synuclein [87].	Overexpression of α-synuclein activates the CaN-NFAT pathway in cell lines and dopaminergic neurons; inhibition of this pathway protects dopaminergic neurons against α-synuclein-mediated toxicity [76]. Inhibition of CaN moves the α-synuclein-induced CaM-CaN cascade to a protective mode. This may have therapeutic implications for the treatment of PD [88].
**(SK) channels**	Neurotransmitter release [89].	Lower expression in PD models [91].	SK channel inhibition protects nigral dopaminergic neurons and improves motor performance in PD model [91]. SK channel activation has protective effects [89].
**Glutamate decarboxylase**	Decarboxylation of L-glutamic acid to GABA [93].	ND	Improvement in motor function in PD patients after adeno-associated virus (AAV)-glutamic acid decarboxylase (GAD)- gene therapy [93].
**NOS**	Learning, memory, neurogenesis	High levels of nNOS and iNOS in the substantia nigra and striatum of PD patients and PD models [97]. nNOS expression is increased in basal ganglia and in the respiratory burst of circulating neutrophils of PD patients; a significant increase in NO production and protein tyrosine nitration is observed [101].	nNOS inhibitor protects neuronal cells against MPTP-induced neurotoxicity in animal models [99,100]. nNOS knockout mice are more resistant to MPTP-induced neurotoxicity compared with wild-type animals [98].

ND—not determined.

## Data Availability

Not applicable.

## Abbreviations

A2AR, adenosine A2A receptor; [Ca^2+^]_i_, intracellular Ca^2+^ concentration; CaM, calmodulin; CaMBPs, calmodulin binding proteins; CaMKII, calmodulin dependent protein kinase 2; CaN, calcineurin; DA, dopaminergic; DLB, dementia with Lewy bodies; ER, endoplasmic reticulum; GPCR, G protein coupled receptors; IP_3_R, inositol-1,4,5-tris-phosphate receptor; L-DOPA, l-3,4-dihydroxyphenylalanine; LBs, Lewy bodies; LRRK2, leucine-rich repeat receptor kinase 2; LTP, long-term potentiation; MCU, Ca^2+^ uniporter; MPTP, 1-methyl-4-phenyl-1,2,3,6-tetrahydropyridine; MPP+, 1-methyl-4-phenylpyridinium; NCX, Na^+^/Ca^2+^ exchanger; NCLX, mitochondrial Na^+^/Ca^2+^ exchanger; NFAT, nuclear factor of activated T-cells; NMDA, N-methyl-D-aspartate; *NMDARs*, *NMDA glutamate* receptors; NCLX, mitochondrial Na^+^/Ca^2+^ exchanger, NOS, nitric oxide synthase; 6-OHDA, 6-hydroxydopamine; ORAI, Ca^2+^-release-activated calcium channel protein; PD, Parkinson’s disease; PMCA, plasma membrane Ca^2+^-ATPase; PP1, protein phosphatase 1; PSD, postsynaptic density; ROS, reactive oxygen species; RyR, ryanodine receptor; SERCA, endoplasmic reticulum Ca^2+^-ATPase; SOC channel, store operated Ca^2+^ channel; SOCE, store operated Ca^2+^ entry; STIM, calcium signal transducer; TRP, transient receptor potential.
